# OA30 Genetic analysis of whole exome sequencing in a cohort of children with refractory JIA reveals genetic risk factors for rare juvenile diseases

**DOI:** 10.1093/rap/rkac066.030

**Published:** 2022-09-28

**Authors:** Melissa Tordoff, Samantha Smith, Gillian Rice, Lucy Wedderburn, Kimme Hyrich, Andrew Morris, Tracy Briggs, Wendy Thomson, Stephen Eyre, John Bowes

**Affiliations:** Centre for Genetics and Genomics Versus Arthritis, Manchester, United Kingdom; Centre for Genetics and Genomics Versus Arthritis, Manchester, United Kingdom; Division of evolution and genomic sciences, Manchester, United Kingdom; Infection, Immunity and Inflammation Research and Teaching Department, UCL Great Ormond Street Institute of Child Health, london, United Kingdom; Centre for Adolescent Rheumatology Versus Arthritis at UCL, london, United Kingdom; NIHR Biomedical Research Centre at Great Ormond Street Hospital, london, United Kingdom; Centre for Epidemiology Versus Arthritis, The University of Manchester, Manchester, United Kingdom; National Institute of Health Research Manchester Biomedical Research Centre, Manchester, United Kingdom; Centre for Genetics and Genomics Versus Arthritis, Manchester, United Kingdom; Division of evolution and genomic sciences, Manchester, United Kingdom; Centre for Genetics and Genomics Versus Arthritis, Manchester, United Kingdom; National Institute of Health Research Manchester Biomedical Research Centre, Manchester, United Kingdom; Centre for Genetics and Genomics Versus Arthritis, Manchester, United Kingdom; National Institute of Health Research Manchester Biomedical Research Centre, Manchester, United Kingdom; Centre for Genetics and Genomics Versus Arthritis, Manchester, United Kingdom; Centre for Genetics and Genomics Versus Arthritis, Manchester, United Kingdom; National Institute of Health Research Manchester Biomedical Research Centre, Manchester, United Kingdom

## Abstract

**Introduction/Background:**

Juvenile idiopathic arthritis (JIA) encompasses a group of heterogeneous rheumatic diseases of childhood onset. JIA can result in long term disability and remission is the main goal of treatment. However refractory disease can occur, which is defined as the absence of response to a standard disease therapy. A genetic basis for refractory disease has yet to explored, where deleterious rare variants complicate diagnosis or treatment outcome. This study aimed to investigate, through genetic analysis, whether children with JIA that is refractory carry rare genetic risk factors in genes linked to monogenic diseases.

**Description/Method:**

Whole exome sequencing of 99 children with JIA was performed with the Agilent SureSelect Human All ExonV6 kit. All quality control, variant filtering and annotation was performed in Varseq (version 2.2.1). Variants with a read depth <30 and genotype quality <80 were removed. Rarity and pathogenicity filters were then applied to remove variants with an allele frequency >1% (based on ExAC, gnomAD, gnomAD exome, NHLBI and 1KGp phase 3), classified as benign or likely benign on ClinVar, with a CADD PHRED score <15 and a REVEL score >0.7. Variants were annotated if they appeared in a gene from the primary immunodeficiency PanelApp (Martin et al., 2019), in a gene associated with an arthritis phenotype or in a gene that appeared on a paediatric monogenic gene list. The variants were then classified using ACMG guidelines (Richards et al., 2015) and benign, or likely benign, classified variants were removed.

**Discussion/Results:**

A total of 470 variants were identified and we found that 20 out of the 99 children screened were heterozygous for at least one recognised variant in a gene linked to a monogenic disease. Five of these children carried more than one recognised variant linked to monogenic genes. Here we provide a number of illustrative examples: three genes, ADAR, ATP7B and MVK, were prioritised based on prior evidence of associated disease. The variant p.Pro193Ala (gnomAD allele frequency (GAD) 2.2x10-3) of ADAR has previously been deemed pathogenic in a homozygous or compound heterozygous state for Aicardi-Goutières syndrome. Adenosine deaminases (ADARs) catalyse the hydrolytic deamination of adenosine to inosine in dsRNA and is suggested to act as a suppressor of type 1 interferon-stimulated genes. Within ATP7B, two distinct variants were detected; p.Gln1142His (GAD 1.6x10-5) and p.Ile1148Thr (GAD 4.0x10-5) have previously been reported as pathogenic, in combination with a third variant, for Wilson’s disease and were carried by one individual in this cohort. ATP7B encodes copper-transporting ATPase 2, which supplies copper to ceruloplasmin. Variant p.Val377Ile (GAD 1.6x10-3) of MVK was detected in eight individuals in this cohort, interestingly five of these individuals also carried at least one HLA-DRB1 stop-gained variant. This MVK mutation has been confirmed as pathogenic in a homozygous or compound heterozygous state for mevalonate kinase deficiency. MVK converts mevalonic acid into mevalonate-5-phosphate in the cholesterol synthesis pathway. Additionally, two stop-gained loss of function HLA-DRB1 variants, p.Tyr107Ter and p.Gln125Ter, were detected in five and 20 individuals, respectively, in this cohort. HLA-DRB1 is a recognised susceptibility locus for JIA.

**Key learning points/Conclusion:**

Screening of a cohort of 99 children with JIA that have refractory disease has revealed that individuals carry deleterious variants in genes linked to monogenic forms of disease. These results highlight that the genetic basis for refractory disease needs to be further investigated. Carrying additional genetic risk factors to disease may complicate disease outcome and genetic screening of children with refractory JIA may improve treatment outcome in the future.

